# Principal component-based weighted indices and a framework to evaluate indices: Results from the Medical Expenditure Panel Survey 1996 to 2011

**DOI:** 10.1371/journal.pone.0183997

**Published:** 2017-09-08

**Authors:** Yi-Sheng Chao, Chao-Jung Wu

**Affiliations:** 1 Centre de Recherche du Centre Hospitalier de l’Université de Montréal (CRCHUM), Université de Montréal, Montréal, Québec, Canada; 2 Département d'Informatique, Université du Québec à Montréal, Montréal, Québec, Canada; University of Rijeka, CROATIA

## Abstract

Producing indices composed of multiple input variables has been embedded in some data processing and analytical methods. We aim to test the feasibility of creating data-driven indices by aggregating input variables according to principal component analysis (PCA) loadings. To validate the significance of both the theory-based and data-driven indices, we propose principles to review innovative indices. We generated weighted indices with the variables obtained in the first years of the two-year panels in the Medical Expenditure Panel Survey initiated between 1996 and 2011. Variables were weighted according to PCA loadings and summed. The statistical significance and residual deviance of each index to predict mortality in the second years was extracted from the results of discrete-time survival analyses. There were 237,832 surviving the first years of panels, represented 4.5 billion civilians in the United States, of which 0.62% (95% CI = 0.58% to 0.66%) died in the second years of the panels. Of all 134,689 weighted indices, there were 40,803 significantly predicting mortality in the second years with or without the adjustment of age, sex and races. The significant indices in the both models could at most lead to 10,200 years of academic tenure for individual researchers publishing four indices per year or 618.2 years of publishing for journals with annual volume of 66 articles. In conclusion, if aggregating information based on PCA loadings, there can be a large number of significant innovative indices composing input variables of various predictive powers. To justify the large quantities of innovative indices, we propose a reporting and review framework for novel indices based on the objectives to create indices, variable weighting, related outcomes and database characteristics. The indices selected by this framework could lead to a new genre of publications focusing on meaningful aggregation of information.

## Introduction

An index or composite measure can be used to represent an idea or an outcome. Many of the weighted indices or composite measures are the summation of the products of variables values and equal or variable-specific weights[1]. Although there may be differences in how to these indices are developed, they have been widely used in social science[1–3], health research and biomedical investigation[4, 5]. The construction of weighted or unweighted indices involves several steps, including validation of individual measures that make up the indices, assessment of the variability between subjects, and index scoring[1, 5]. Some differences exist across disciplines or research subjects and thus specialized methods may be preferred[6]. Besides external validity and generalizability, the statistical significance or predictive power between the produced index and external outcomes is also important for wider use or subsequent application to other research topics[1]. For example, the concept of frailty, defined as a geriatric syndrome, has been characterized by indices composed of different sets of multiple indicators, especially weight loss and less grip strength[5, 7, 8]. A variety of frailty indices have been proven useful and statistically significant to predict major outcomes, such as mortality[9], surgical outcomes[10] and occurrence of disability[11].

The generation of weighted or unweighted indices has been important to operationalize abstract subjects or create new research tools. However, the use of indices or composite measures is more prevalent than many may expect. The process of producing indices that are composed of a subset of variables from a database has been embedded in several data processing and analytical methods. For example, principal components (PCs) are the linear combinations of the variables according to principal component analysis (PCA)[12]. Partial least squares (PLS) regression also applies a set of loadings to the original input variables, though different from those obtained from the PCA[12]. Addictive models use multiple functions that aggregate features with potentially dissimilar coefficients to derive new input functions for outcome prediction[12]. Neural networks use original inputs to obtain a number of derived variables that then serve as predictors for the outcomes in multi-layer models[12]. The implicit creation of indices, most likely to be of unequal weights for input variables, makes us curious about whether it is possible to reproduce and fine-tune the process of information aggregation or index generation in these methods.

Combining the conventional view that takes statistical significance as the criteria for the validity of indices and the prevalent use of data aggregation and implicit weighting of input variables, we aim to propose and test a data-driven procedure of “index mining” or a systematic search for optimal variable aggregation. By taking PCA as an example to assign weights to input variables, the procedures to aggregate input variables are according to PCA loadings and a PCA-based method to generate statistically significant mortality indices is developed. After index mining, we also propose a review framework to examine the validity of newly generated indices according to the differences we identify between the data-driven approach and prevalent theory-based index-generating methods.

## Methods

**Fig 1:**
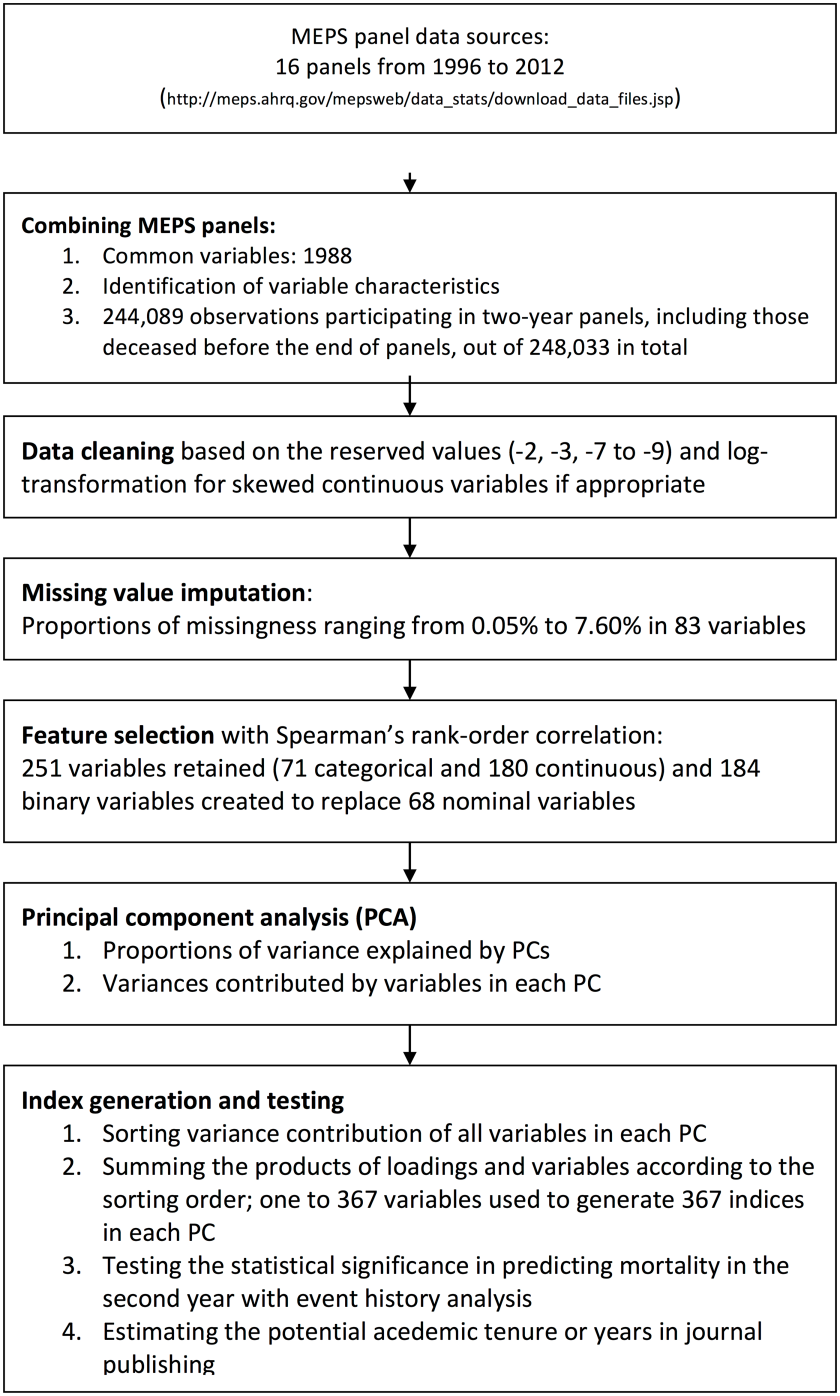
A flow chart of data linkage, data processing, feature selection, principal component analysis and index generation with the Medical Expenditure Panel Survey (MEPS) 1996 to 2012.

This secondary data analysis study was approved by the ethics committee of the Centre hospitalier de l'Université de Montréal. We generated weighted indices with the variables from the first years of the two-year Medical Expenditure Panel Survey (MEPS) panels according to PCA loadings to predict mortality in the second years (see Fig 1 for the flowchart). First, we conducted PCA with year-one variables to obtain the loadings to construct each PC. Second, we sorted the input variable by the absolute values of loadings in each PC to generate weighted indices. The input variables with larger absolute values of loadings were summed first for each PC. Third, the indices were the sums of the products of input variables and PCA loadings. Fourth, the statistical significance and deviance of each index to predict mortality in the second years was extracted from the results in discrete-time event history analyses[13].

### Data sets

This study analyzed the 16 longitudinal panels released from the MEPS that were conducted annually among civilian non-institutionalized population to produce nationally representative statistics since 1996 in the United States[14]. Each panel lasted for two years and consisted of five rounds of data collection[15]. Only year-one variables were used for PCA to predict mortality in the second years.

### Data linkage and processing

The 16 longitudinal two-year panels of the MEPS were pooled by variable names common to all panels[16]. There were 1989 common year-one variables across 16 panels (panels beginning throughout 1996 and 2011, see S1 Table for the list of variables and their characteristics). Only subjects participating throughout the two-year panels were retained in the data set, in addition to those deceased before the end of the two-year panels. Administrative variables and the variables that were used to flag certain circumstances in data gathering were not used for PCA. To avoid overlapping information and increase the computational feasibility, the 789 variables containing individual information in the first years of the two-year panels were retained for further variable selection and analysis.

Reserved values that identified specific responses across all variables were recoded according to the MEPS codebooks: -2 recoded to the same answers in previous rounds, -1 to inapplicability and others to missing values (-3, -7, -8, and -9 for “no data in round”, “refused”, “do not know”, and “not ascertained” respectively); see S1 Table for the percentages of observations in these categories of the variables).[16]. The skewness of continuous variables was evaluated without adjusting for survey design. Log transformation was applied if the skewness of log-transformed variables were less than original variables[17].

### Feature selection with Spearman’s rank order correlation

This study first selected features with a correlation-based method proposed for the purpose of removing redundant variables and increasing computational feasibility[18, 19]. The data redundancy might be created for the ease of survey implementation or data labeling. For example, different sources of income were separately asked and total income was the sum of incomes from all sources[20]. The levels of education might be presented in years spent in school or types of highest grade completed[20] (See S1 Table for details in variable names and labels).

Spearman’s rank-order correlation was used to create a correlation matrix of all variables, categorical or continuous[18, 19]. For each pair of variables in the correlation analysis, the subjects were dropped if there were any missing values in these two variables. The threshold for redundancy was Spearman’s rank correlation coefficient greater than 0.9[21]. There were 251 variables left for further analysis (see Fig 1 for the flowchart). The proportions of missingness ranged from 0% to 7.18%, median 0.18% among 83 variables with any missing values. Sixty-eight of the retained variables were categorical and 15 were continuous. After variable selection, missing values in all variables were imputed with the multivariate imputation by chained equations[22].

Of the 71 categorical variables, three ordinal variables that ranked poverty categories (povcaty1), difficulty in using fingers to grasp (fngrdf1), and a summary measure of vision impairment (vision2) were not transformed. Other 68 nominal variables were replaced with 184 binominal variables. This led to 367 variables available for PCA and 15 variables used for personal identification and control for survey design.

### Principal component analysis

PCA, PC for principal component, was proven useful for dimension reduction or data pre-processing[23]. Although there were other choices of PCA[23–25] or similar data techniques[12, 26], the choices of dimension reduction methods applicable to survey design were limited[27]. We considered linear PCA as the optimal and feasible option in consideration of complex survey design[28]. Before PCA, each variable was centered to zero and scaled to unit variance. PCA was conducted with the 367 variables while adjusting for survey design[27]. The PC values were predicted for each subject.

### PCA-based index generation

The indices were generated according to PCA loadings. Each PC was a linear combination of all input variables and could be seen as a weighted sum of all variables after input variables being catered and scaled[29]. The number of PCs was the same as the number of input variables, denoted by *N*. In Eq 1, a PC, specified with a subscript *pc*, was the sum of all input variables, denoted by *x*, weighted by PC-specific loadings, denoted by *L*.

$$PC_{pc}=\sum_{i=1}^{N}L_{i}x_{i}$$ (1)

The process of PCA-based index generation was described as follows. The first index of each PC, denoted by *Index*_*pc*.*n*_, was the product of the leading variable, in terms of absolute values of loadings, and its PC-specific loading, denoted by *L*_*i*_*x*_*i*_ while *pc* referring the PC that was used to produce indices, *n* specifying the numbers of input variables required for the index in Eq 2, and *n* equalling one. The second one was the sum of the products of the first two leading variables weighted by PC-specific loadings, denoted by $\sum_{i=1}^{2}L_{i}x_{i}$. By repeating the same procedure, we include all variables weighted by loadings in each PC and the last index in each PC was the same as the PC value. There were 367 weighted indices generated for each PC, 134,689 for 367 PCs in total.

$$Index_{pc.n}=\sum_{i=1}^{n}L_{i}x_{i}$$ (2)

### Descriptive survival analysis

The outcome of interest was mortality in the second years. The survival function of the MEPS interviewees was estimated with Kaplan-Meier method[30] and adjusted for survey design[28] by months in the second years of the panels. We tested the differences in survival functions by sex and race/ethnicity.

The deaths in the second years were modelled in four three-month periods or quarters: January to March, April to June, July to September, and October to December. Each individual was duplicated for each quarter if they remained alive. For example, an individual that survived throughout the second years of the MEPS panels would have four data entries representing four quarters. Each data entry was labelled alive. If someone died in the third quarter, July to September, they would only have three observations for non-existence in the fourth quarter and the third entry was labelled dead.

#### Discrete-time event history analysis

The survival of the MEPS participants in the second year of each panel was modelled with discrete-time event history analysis for the violation of the proportional hazard assumption in Cox model by generated indices[31]. We tried with first few indices and found that the proportional-hazard assumption for the Cox-proportional regression model might not hold for most indices[31]. In unadjusted models, deaths in each discrete time periods, quarters, were predicted with each generated index, time and interaction between index and quarters (see S1 Equation for details). In adjusted models, age, sex and races were added as independent variables. Ages in years were calculated based on the birth and interview dates. Sex included male and female. Races were white, black, American Indians or Alaska natives, native Hawaiian or Pacific islanders, and multiple races. Event history analysis[13] for binominal outcome, mortality, was conducted with the adjustment of complex survey design in the MEPS data sets with *survey* package[28] available in R (v3.2.2)[32] and RStudio environment (0.99.903)[33].

#### Proposed publication cycles for weighted indices

Because of the large number of significant indices generated from the MEPS data alone, we would like to estimate the impact of new indices on academic publishing and knowledge translation, assuming three steps required within three-month periods of index mining: generation of weighted indices, formation of theories and methods, and comparisons across databases (Table 1). In each step, a manuscript for publication was drafted and three manuscripts generated for one index. For each significant index, a researcher could use it to publish one article every month and four articles per year. This might help to secure academic tenure by publishing four innovative indices per year or 12 related articles annually. The estimated impact on academic tenure was the number of years that a researcher could maintain this pace of publication, estimated by dividing the number of significant indices by four.

**Table 1:** Proposed publication cycle for weighted indices.

		Repeat until running out of indices for desired outcomes		
Stages in publication cycles	Preparation	1^st^ month	2^nd^ month	3^rd^ month
**Objective**	Search for all significant indices	Generate theories or hypotheses to introduce new indices	Publish PCA-based indices	Validate the published index
**Activities**	Select a database and a target outcome Generate PCA-based indices Significance testing regarding one particular outcome Summarize the number of significant indices	Select a significant index consciously or randomly Create index names and attach new theories or hypotheses Publish new theories	Use the statistically significant weighted index to support new theories or hypotheses	Emphasize the importance and significance of the index by demonstrating its significant role in other outcomes, other data sources, other subpopulations, other contexts and so on.

#### Estimated time of journal publishing

The average number of articles published in an academic journal was about 64 to 68 annually in 2012, 1.8 to 1.9 million articles by 28,100 active journals[34]. We assumed that there was a journal focusing on publishing innovative and significant indices. The expected time of journal publishing in numbers of years was estimated through dividing the total number of significant weighted indices by 66.

## Results

### Survival rates by months in the second years of the MEPS panels

**Fig 2:**
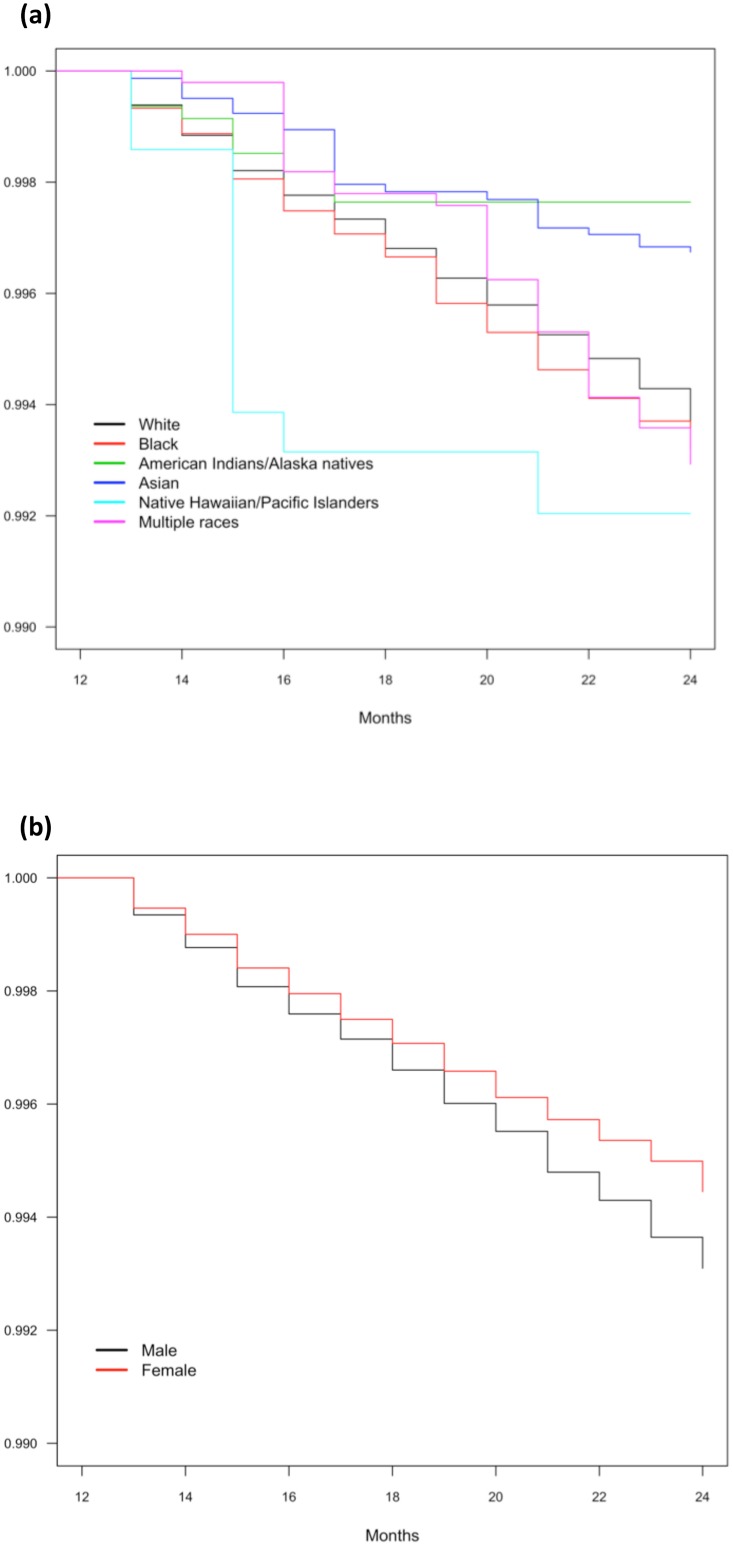
The Kaplan-Meier survival curves of the interviewees in the second years of the MEPS panels. (a) The Kaplan-Meier survival curves by sex. Chi-square = 12.23, p < 0.001. (b) The Kaplan-Meier survival curves by races. Chi-square = 27.46, p < 0.001.

There were 244,089 individuals surveyed throughout the two-year panels in the first to 16^th^ MEPS panels. There were 237,832 surviving the first years of panels. This represented 4.5 billion civilians in the United States, of which 0.62% (95% CI = 0.58% to 0.66%) died in the second years of the panels. The demographic characteristics were listed in Table 2. The proportions of two sexes and white or non-white races were not statistically different across the MEPS panels (p = 1 and 0.24 respectively in Fig 2). The proportions of dying in the second years of the MEPS panels were not the same across panels (p < 0.01). The Kaplan-Meier survival curves by sex and races were shown in Fig 2. The survival curves by months in the second years were significantly different across sex and races (p < 0.001 for both).

**Table 2:** The characteristics of the interviewees in the first to 16^th^ Medical Expenditure Panel Survey.

Panels	Begin years	Sample sizes (n)			Female (%)		Races (%)												Died in the 2nd years of panels (%)	
		Unweighted	Weighted	(95% CI)		(95% CI)	White	(95% CI)	Black	(95% CI)	American_Indians/Alaska_natives	(95% CI)	Asian	(95% CI)	Native_Hawaiian/Pacific_islanders	(95% CI)	Multiple_races	(95% CI)		(95% CI)
**1**	1996	18,847	260,676,916	(243,877,393 to 277,476,439)	51.29%	(50.59% to 52.00%)	81.77%	(80.12% to 83.43%)	13.09%	(11.65% to 14.53%)	1.30%	(0.86% to 1.75%)	3.76%	(2.99% to 4.52%)	0.00%	(0.00% to 0.00%)	0.08%	(0.02% to 0.13%)	0.14%	(0.08% to 0.21%)
**2**	1997	11,917	266,865,458	(238,549,994 to 295,180,923)	51.21%	(50.39% to 52.03%)	82.76%	(80.68% to 84.84%)	13.04%	(11.06% to 15.02%)	0.91%	(0.59% to 1.22%)	3.29%	(2.38% to 4.20%)	0.00%	(0.00% to 0.00%)	0.00%	(0.00% to 0.00%)	0.66%	(0.49% to 0.82%)
**3**	1998	9,704	268,961,612	(235,532,351 to 302,390,873)	51.25%	(50.29% to 52.21%)	81.41%	(79.07% to 83.75%)	13.15%	(11.02% to 15.28%)	0.59%	(0.35% to 0.83%)	4.85%	(3.64% to 6.07%)	0.00%	(0.00% to 0.00%)	0.00%	(0.00% to 0.00%)	0.69%	(0.50% to 0.88%)
**4**	1999	12,833	271,359,560	(223,667,123 to 319,051,996)	51.19%	(50.43% to 51.96%)	82.34%	(79.88% to 84.80%)	13.20%	(10.63% to 15.78%)	1.11%	(0.52% to 1.70%)	3.34%	(2.58% to 4.10%)	0.00%	(0.00% to 0.00%)	0.00%	(0.00% to 0.00%)	0.65%	(0.45% to 0.86%)
**5**	2000	10,000	275,825,314	(228,649,135 to 323,001,494)	51.28%	(50.30% to 52.27%)	83.05%	(80.68% to 85.41%)	12.95%	(10.61% to 15.29%)	0.55%	(0.30% to 0.79%)	3.46%	(2.62% to 4.30%)	0.00%	(0.00% to 0.00%)	0.00%	(0.00% to 0.00%)	0.68%	(0.50% to 0.87%)
**6**	2001	20,328	280,432,464	(249,937,923 to 310,927,005)	51.17%	(50.54% to 51.80%)	81.13%	(79.48% to 82.79%)	12.27%	(10.64% to 13.90%)	1.02%	(0.67% to 1.37%)	3.83%	(3.05% to 4.61%)	0.35%	(0.11% to 0.59%)	1.40%	(1.10% to 1.69%)	0.65%	(0.53% to 0.77%)
**7**	2002	15,513	282,724,249	(253,127,157 to 312,321,342)	51.10%	(50.34% to 51.87%)	81.48%	(79.90% to 83.07%)	12.33%	(10.85% to 13.81%)	0.80%	(0.53% to 1.07%)	3.86%	(3.10% to 4.63%)	0.27%	(0.13% to 0.40%)	1.26%	(0.99% to 1.53%)	0.66%	(0.50% to 0.82%)
**8**	2003	15,549	285,244,087	(254,060,021 to 316,428,152)	51.08%	(50.45% to 51.70%)	81.01%	(79.26% to 82.75%)	12.36%	(10.70% to 14.01%)	0.64%	(0.36% to 0.93%)	3.92%	(3.18% to 4.67%)	0.33%	(0.15% to 0.52%)	1.73%	(1.32% to 2.15%)	0.66%	(0.50% to 0.81%)
**9**	2004	15,398	287,469,219	(262,449,744 to 312,488,694)	51.07%	(50.31% to 51.82%)	80.20%	(78.25% to 82.15%)	12.49%	(10.75% to 14.23%)	0.83%	(0.49% to 1.17%)	4.26%	(3.40% to 5.13%)	0.38%	(0.17% to 0.59%)	1.84%	(1.48% to 2.20%)	0.62%	(0.47% to 0.77%)
**10**	2005	14,961	290,237,146	(264,335,686 to 316,138,607)	51.08%	(50.32% to 51.83%)	80.38%	(78.49% to 82.28%)	12.41%	(10.80% to 14.01%)	0.78%	(0.41% to 1.15%)	4.33%	(3.47% to 5.19%)	0.35%	(0.08% to 0.62%)	1.75%	(1.37% to 2.14%)	0.63%	(0.46% to 0.80%)
**11**	2006	15,871	292,567,761	(270,395,864 to 314,739,659)	51.09%	(50.42% to 51.76%)	80.14%	(78.44% to 81.85%)	12.38%	(10.99% to 13.78%)	0.91%	(0.52% to 1.30%)	4.37%	(3.52% to 5.22%)	0.45%	(0.19% to 0.71%)	1.74%	(1.37% to 2.10%)	0.62%	(0.48% to 0.76%)
**12**	2007	11,965	295,618,849	(275,773,974 to 315,463,724)	50.97%	(50.17% to 51.76%)	80.43%	(78.37% to 82.50%)	12.35%	(10.56% to 14.13%)	0.79%	(0.45% to 1.12%)	4.30%	(3.46% to 5.13%)	0.21%	(0.11% to 0.31%)	1.93%	(1.47% to 2.39%)	0.62%	(0.44% to 0.80%)
**13**	2008	17,510	297,983,418	(281,865,082 to 314,101,755)	50.96%	(50.22% to 51.70%)	79.93%	(78.15% to 81.71%)	12.37%	(10.98% to 13.76%)	0.78%	(0.42% to 1.14%)	4.57%	(3.74% to 5.41%)	0.25%	(0.11% to 0.40%)	2.09%	(1.67% to 2.51%)	0.65%	(0.48% to 0.82%)
**14**	2009	15,642	300,419,079	(282,428,580 to 318,409,578)	50.94%	(50.26% to 51.63%)	79.86%	(78.09% to 81.62%)	12.54%	(10.97% to 14.11%)	0.90%	(0.45% to 1.35%)	4.67%	(3.79% to 5.55%)	0.29%	(0.15% to 0.44%)	1.74%	(1.37% to 2.11%)	0.67%	(0.48% to 0.86%)
**15**	2010	13,977	303,027,202	(286,337,440 to 319,716,964)	51.21%	(50.42% to 51.99%)	79.59%	(77.64% to 81.55%)	12.32%	(10.76% to 13.87%)	0.81%	(0.30% to 1.32%)	4.97%	(3.96% to 5.99%)	0.61%	(0.34% to 0.88%)	1.70%	(1.35% to 2.05%)	0.63%	(0.46% to 0.81%)
**16**	2011	17,817	305,055,352	(287,349,122 to 322,761,583)	51.16%	(50.46% to 51.86%)	79.91%	(78.01% to 81.82%)	12.39%	(11.01% to 13.77%)	0.00%	(0.00% to 0.00%)	5.16%	(4.00% to 6.32%)	0.00%	(0.00% to 0.00%)	2.53%	(2.11% to 2.95%)	0.64%	(0.49% to 0.79%)
**All**	All panels	237,832	4,564,467,688	(4,394,590,562 to 4,734,344,813)	51.12%	(50.92% to 51.33%)	80.92%	(80.17% to 81.68%)	12.59%	(11.90% to 13.28%)	0.79%	(0.66% to 0.92%)	4.20%	(3.87% to 4.54%)	0.22%	(0.17% to 0.27%)	1.27%	(1.18% to 1.37%)	0.62%	(0.58% to 0.66%)

Note: the proportions by sex and white race are not statistically different by the panels (p = 1 and 0.24 respectively). The proportions of dying in the second years of the MEPS panels are different (p < 0.01).

### Principal components and survival

**Fig 3:**
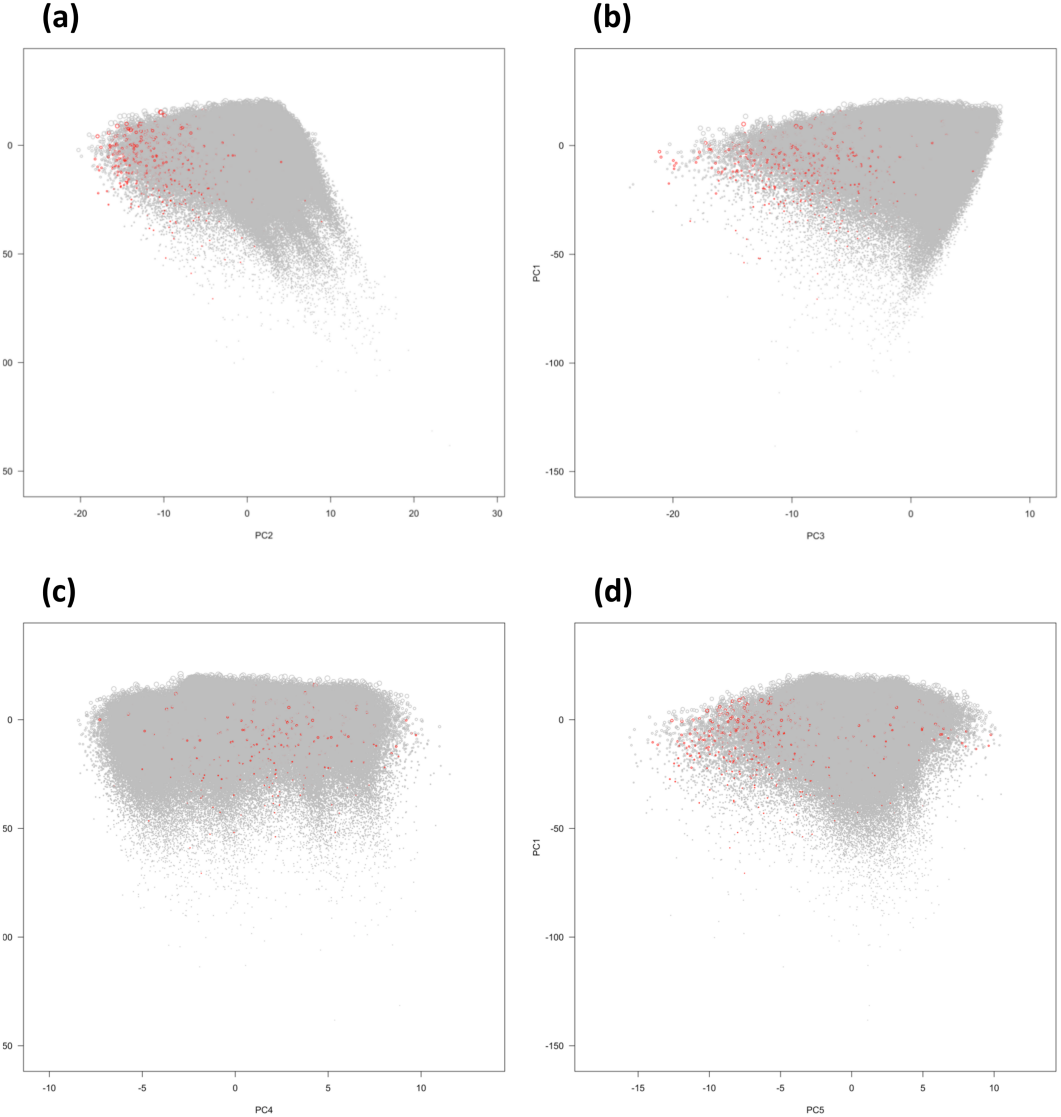
The distributions of those dying in the second years of the MEPS panels by principal components. (a) The distribution of those dying by first and second principal components (PC1 and PC2). (b) The distribution of those dying by first and third principal components (PC1 and PC3). (c) The distribution of those dying by first and fourth principal components (PC1 and PC4). (d) The distribution of those dying by first and fifth principal components (PC1 and PC5). Note: red circles: those dying in the second years of the MEPS panels; gray circles: those surviving throughout the MEPS panels.

The leading variables contributing the most to the first five PCs were listed in S2 Table. The PC values of those dying in the second years of the MEPS panels were plotted against those surviving throughout the panels in Fig 3. Those dying in the second years did not seem to evenly distribute across the first five PCs, especially in PC2 and PC3. In Fig 3a and 3b, those dying in the second years seemed to be associated with lower PC2 and PC3. Taking PC1 and PC2 as examples, the coefficients to predict the mortality risks obtained from even history analyses were shown in Tables 3 and 4. PC1 was not significant in the unadjusted model that only accounted for time, quarters in the second years, and interactions between PC1 and time (p = 0.78). However, in the adjusted model that added age, sex and races as predictors, PC1 was significantly associated with mortality risk (p < 0.001).

**Table 3:** Coefficients of the first principal component to predict mortality in the second years of the MEPS panels.

	PC1			PC2			PC3		
	coef	(95% Cis)	p	coef	(95% Cis)	p	coef	(95% Cis)	p
**PCs**	-0.030	(-0.234 to 0.175)	0.78	-0.309	(-0.329 to -0.289)	0.78	-0.242	(-0.260 to -0.223)	<0.001
**Quarters in the second years of MEPS panels**									
**1**	-6.287	(-8.722 to -3.853)	<0.001	-7.476	(-7.670 to -7.282)	<0.001	-6.906	(-7.062 to -6.750)	<0.001
**2**	-6.512	(-9.243 to -3.782)	<0.001	-7.694	(-7.949 to -7.440)	<0.001	-7.227	(-7.418 to -7.037)	<0.001
**3**	-6.399	(-8.985 to -3.812)	<0.001	-7.279	(-7.478 to -7.080)	<0.001	-6.989	(-7.174 to -6.803)	<0.001
**4**	-6.459	(-9.133 to -3.785)	<0.001	-7.363	(-7.558 to -7.168)	<0.001	-6.964	(-7.142 to -6.786)	<0.001
**Interactions terms**									
**PC1:Q2**	0.005	(-0.310 to 0.319)	0.98	-0.002	(-0.033 to 0.030)	0.91	-0.019	(-0.047 to 0.009)	0.18
**PC1:Q3**	0.007	(-0.301 to 0.315)	0.97	0.037	(0.004 to 0.071)	0.03	0.003	(-0.029 to 0.035)	0.87
**PC1:Q4**	0.008	(-0.307 to 0.324)	0.96	0.031	(0.001 to 0.062)	0.04	0.019	(-0.011 to 0.050)	0.21

**Table 4:** Coefficients of the first principal component and demographics to predict mortality in the second years of the MEPS panels.

	PC1			PC2			PC3		
	coef	(95% Cis)	p	coef	(95% Cis)	p	coef	(95% Cis)	p
**PCs**	-0.030	(-0.038 to -0.021)	<0.001	-0.219	(-0.243 to -0.194)	<0.001	-0.100	(-0.128 to -0.072)	<0.001
**Quarters in the second years of MEPS panels**									
**1**	-10.136	(-10.521 to -9.751)	<0.001	-8.983	(-9.369 to -8.597)	<0.001	-10.027	(-10.446 to -9.608)	<0.001
**2**	-10.351	(-10.743 to -9.959)	<0.001	-9.201	(-9.625 to -8.777)	<0.001	-10.391	(-10.865 to -9.918)	<0.001
**3**	-10.227	(-10.616 to -9.838)	<0.001	-8.730	(-9.101 to -8.358)	<0.001	-10.085	(-10.521 to -9.648)	<0.001
**4**	-10.278	(-10.660 to -9.896)	<0.001	-8.819	(-9.172 to -8.465)	<0.001	-10.013	(-10.441 to -9.585)	<0.001
**Age (years)**	0.082	(0.078 to 0.087)	<0.001	0.050	(0.045 to 0.054)	<0.001	0.073	(0.067 to 0.078)	<0.001
**Female**	-0.496	(-0.619 to -0.374)	<0.001	-0.628	(-0.753 to -0.502)	<0.001	-0.475	(-0.597 to -0.352)	<0.001
**Races (baseline: white)**									
**Black**	0.318	(0.150 to 0.486)	<0.001	0.396	(0.236 to 0.556)	<0.001	0.598	(0.437 to 0.759)	<0.001
**American_Indians/Alaska_natives**	-0.472	(-1.315 to 0.372)	0.27	-0.655	(-1.523 to 0.212)	0.14	-0.332	(-1.170 to 0.507)	0.44
**Asian**	-0.360	(-0.830 to 0.111)	0.13	-0.253	(-0.735 to 0.229)	0.30	-0.096	(-0.564 to 0.371)	0.69
**Native_Hawaiian/Pacific_islanders**	0.671	(-0.642 to 1.983)	0.32	0.719	(-0.581 to 2.018)	0.28	0.895	(-0.474 to 2.264)	0.20
**Multiple races**	0.692	(0.167 to 1.217)	0.01	0.507	(-0.031 to 1.045)	0.06	0.789	(0.261 to 1.318)	<0.01
**Interactions terms**									
**PC1:Q2**	0.006	(-0.009 to 0.021)	0.43	-0.003	(-0.041 to 0.035)	0.89	-0.028	(-0.070 to 0.013)	0.18
**PC1:Q3**	0.009	(-0.005 to 0.023)	0.21	0.044	(0.005 to 0.082)	0.03	0.005	(-0.043 to 0.052)	0.84
**PC1:Q4**	0.011	(-0.003 to 0.025)	0.12	0.036	(0.000 to 0.072)	0.05	0.030	(-0.015 to 0.076)	0.19

**Fig 4:**
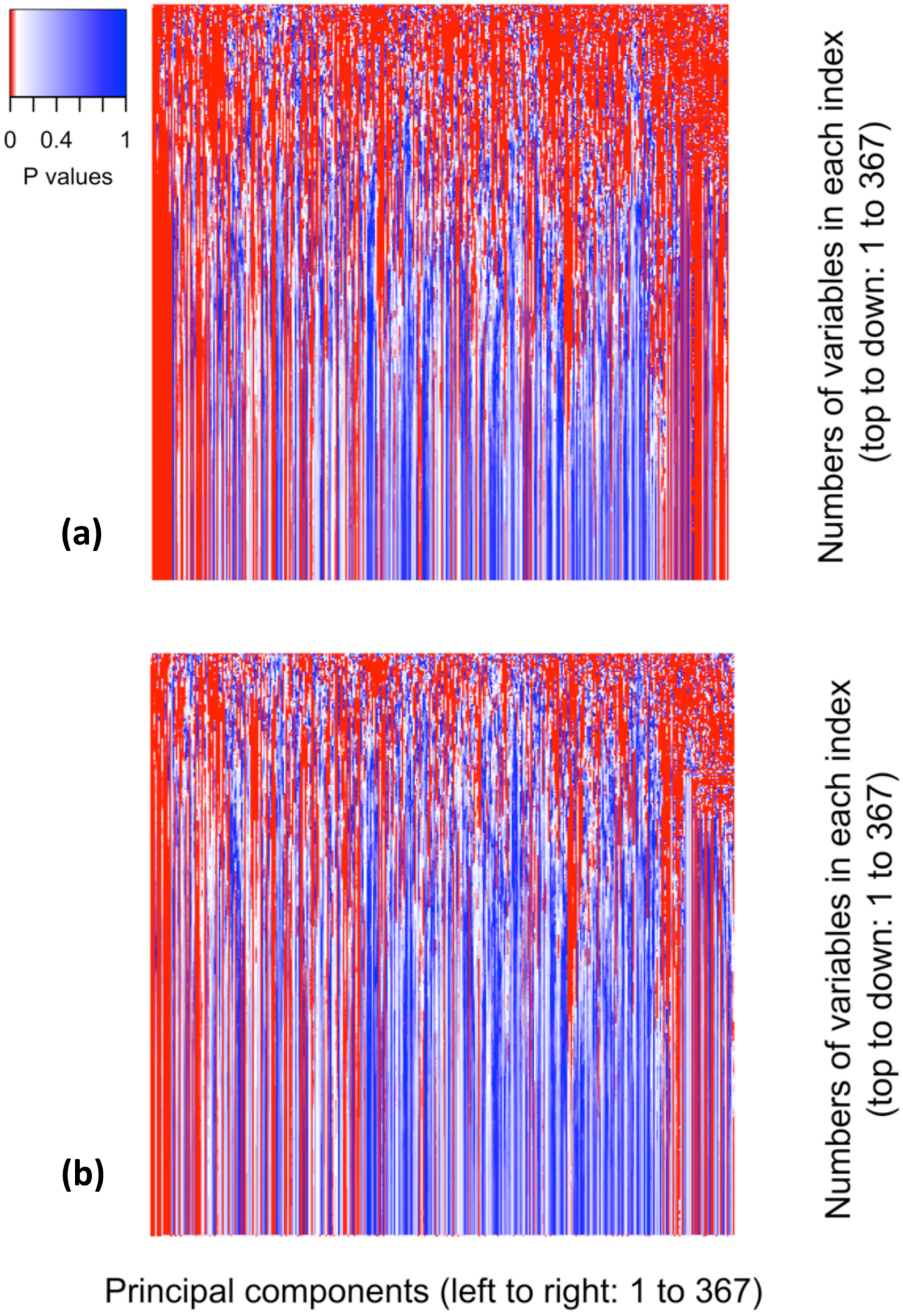
The p values of all PCA-based weighted indices regarding the prediction of mortality risk. (a) P values for 134689 PCA-based indices regarding mortality risk in models that take time (in quarters) and interactions between indices and time. (b) P values for 134689 PCA-based indices regarding mortality risk in models that take age, sex, races, time (in quarters) and interactions between indices and time.

The p values of all 134,689 weighted indices with or without the adjustment of age, sex and races were plotted in Fig 4. Statistical significance represented by red color prevailed both graphs. In Table 5, the numbers of weighted indices were categorized by the numbers of variables composing the indices. The number of input variables that significantly predicted the mortality probability in the second years was 208, 56.68% of all input variables, in both adjusted and unadjusted models. The proportions of significant indices diminished with the number of input variables, from one to, 30, 70 and 367. However, there were still large numbers of significant indices in both unadjusted and adjusted models. All weighted indices composed both significant and insignificant input variables and none of them could be uniquely constructed with significant or insignificant variables.

**Table 5:** Summaries of the significance (p<0.05) of all PCA-based weighted indices.

	Unadjusted and adjusted models			Adjusted models			Unadjusted models		
	Insignificant indices (n)	Significant indices (n)	% of significant indices	Insignificant indices (n)	Significant indices (n)	% of significant indices	Insignificant indices (n)	Significant indices (n)	% of significant indices
**1 variable**	159	208	56.68%	149	218	59.40%	78	289	78.75%
**2 to 30 variables**	5,482	5,161	48.49%	4,863	5,780	54.31%	3,115	7,528	70.73%
**31 to 70 variables**	8,191	6,489	44.20%	7,404	7,276	49.56%	5,006	9,674	65.90%
**71 variables or more**	79,696	28,945	26.64%	76,574	32,067	29.52%	65,790	43,209	39.64%
**All**	93,528	40,803	30.37%	88,990	45,341	33.75%	73,989	60,700	45.07%

### Impact on academic research and journal publishing

Following the proposed publication cycles in Table 1, the 40,803 weighted indices in the both models could lead to 10,200 years of academic tenure for individual researchers or 618.2 years of publishing for journals with annual publication of 66 articles. If young or new researchers were wary of the publication of complicated indices and would like to use significant ones composed of more than one and less than 30 variables[8], the 5,161 indices could lead to 1290.25 years of academic tenure or 78.19 years in journal publishing. For certain research topics, about which 70-item index might be acceptable[35], the volume of significant indices might be sufficient for 1,622.25 years of academic tenure and 98.32 years of journal publication due to 6,489 significant indices composing 31 t o70 input variables.

## Discussion

There are opportunities and challenges identified from the data-driven index mining process. There are several important points learned from the process and results of data-driven index mining. First, the number of significant innovative indices composing multiple input variables is large and the proportion is beyond the probability that we may expect, one out of 20, if aggregating variables according to PCA loadings. In addition to PCs that are often used in PC regression and other models[12], we find that aggregating input variables according to the order of absolute values of PCA loadings is an alternative to search for composite measures or indices significantly predicting outcomes. Based on the large number of alternative indices to predict mortality based on this data-driven method, we suspect the process of traditional or theory-based index generation may not be optimal. For example, the frailty index of input variables assigned with unequal weights derived from neural networks predict adverse outcomes better than that of input variables assigned with equal weights[36]. A systematic approach to review new and innovative indices is required to obtain and select useful indices.

Second, all of the significant indices compose input variables of unequal weights. This contrasts the usual practice of assigning equal weights to all input variables[6, 8]. In addition to assigning equal or PCA-based weights to input variables, there are other methods to assign weights that have been rigorously tested based on the theories or quantitative evidence. For example, the 10-year risk of cardiovascular disease is calculated based on the regression model that predict the occurrence of cardiovascular disease[37]. The regression coefficients that are unequal are regarded as the weights for input variables[37]. The human development index is the multiplicative products of three dimensions regarding health, education and standard of living[38].

In this empirical study, using equal weights in most indices is not the best method to aggregate information or augment signal in this data set. Compared to the loadings obtained from PCA in S2 Table, the indices using equal weights for each input variable will not be optimal in terms of variance maximization. However, there are at least two occasions in PCA, in which the loadings of the input variables are similar. One is that the input variables are highly correlated and summing them with equal weights maximizes the variance of one of the PCs. However, this is to sum variables that resemble each other. This can be a solution to the problem of collinearity in regression models[12]. Unfortunately, this also means the input variables do not provide information much different from each other. This type of indices may be reducible to one or two of the input variables. The other situation for homogenous loadings in one PC is that these variables have very low between-variable correlations, such as the first few leading variables in the PC1 in S2 Table. We think this would be another occasion to apply equal or homogenous weights to input variables. However, whether uncorrelated information from two measures can be summed to represent a concept may need further justification.

Third, using equal weights is a strong and possibly arbitrary assumption for the relationships between input variables and their predictive power. In Eq 1 shown below, the coefficient of the index (*β*_*index*_) regarding a hypothetical outcome (*y*) can be transmitted to all input variables (*x*_*i*_)[29]. The input variables included in the index are subsequently assigned with the regression coefficients, *β*_*index*_ *w*_*i*_ for each *x*_*i*_.

$$\begin{array}{ll} y & =\beta_{0}+\beta_{index}Index+\varepsilon \\ & =\beta_{0}+\sum_{i=1}^{N}\beta_{index}w_{i}x_{i}+\varepsilon \end{array}$$ (3)

This means that the weighting scheme (*w*_*i*_) links the relative scales of predictive power of all input variables and assumes the regression coefficients of the input variables regarding the outcome should not be estimated individually. The coefficients should be set collectively (*β*_*index*_ *w*_*i*_ for each *x*_*i*_). For another outcome, the same restriction applies and the actual coefficients of the input variables simultaneously change in the same relative scales, as a new *β*_*index*_ for all input variables regarding the new outcome.

For indices created solely to represent concepts or abstracts ideas that cannot be measured with singles variables, the pre-determined scales or weights for all input variables may be justifiable. For example, there are proxy indices that are generated to represent functionality[39], emotional well-being and quality of life[40]. However, the existence of some indices are partly justified by significant associations with major outcomes, such as mortality[9] and surgical outcomes[10]. They are more frequently used as outcome predictors than proxy measures of abstract ideas.

For indices frequently used as proxy predictors, the restriction on the relationships and relative scales of all input variables by enforcing an index coefficient may not be ideal. Questions, like why not directly use single variables as predictors to obtain variable coefficients (*β*_*i*_ for each *x*_*i*_) if there are sufficient numbers of sample sizes, how to interpret the composite coefficients (*β*_*index*_*w*_*i*_ for each *x*_*i*_) derived from the index coefficients, how much of the outcome variability can be explained by each input variable and how the outcome may change with the alteration of one input variable if controlling for another input variable of the index, will not be easy to answer. If these questions are the major concern for researchers, using indices as proxies to predict outcomes may not be ideal.

Fourth, for the indices used as predictors, equal or PCA-based weights can be further improved using methods that combine the information from outcomes. Besides PCA, there are other data or estimation methods to take both input variables and outcomes into consideration and generate weighted composite measures, such as partial least squares (PLS) transformation[29]. By applying the PLS projection, the weighting schemes can be searched in consideration of both outcomes and independent variables. We notice that there are many indices that are used heavily as proxy measures and generated without considering outcomes[1, 4–6, 9, 10]. In fact, there are many unexplored alternatives that can be used to determine the optimal or ideal weighting scheme for input variables. Two of the alternatives are subset selection and shrinkage methods that search the set of coefficients optimized for outcomes based on model fit criteria, such as mean square errors or Bayesian information criterion[12]. However, this approach is not applicable for our data that requires the adjustment of survey design.

Fifth, our results support that weak classifiers can be combined to form stronger classifiers[41]. We find that the there is no single PC-based index composing only the input variables that significantly predict mortality. Those insignificant input variables can be combined to obtain new insight toward the prediction of mortality. This can be partly due to the information gain from the weak classifiers that supplement the information of strong classifiers[41]. The use of insignificant or weak predictors in the formation of new indices may need to be systematically explored and should be put more attention.

Lastly, the publication of new indices may help to secure academic tenure and journal publication. The is because of the fact that the number of publications is significantly associated with tenure decisions[42]. The large number of significant indices can help researcher to generate hypotheses or theories in order to augment their publication portfolios and secure academic tenure. For journals, this suggests it is possible to maintain the publication volume with research articles using significant indices. However, the estimation about the numbers of publications still needs to be tested in real world. We observe that adjusting the numbers of input variables in an index by a multiple of ten for publication seems to be well accepted for theory-based indices[35, 43, 44]. This publication strategy should be tried first.

**Fig 5:**
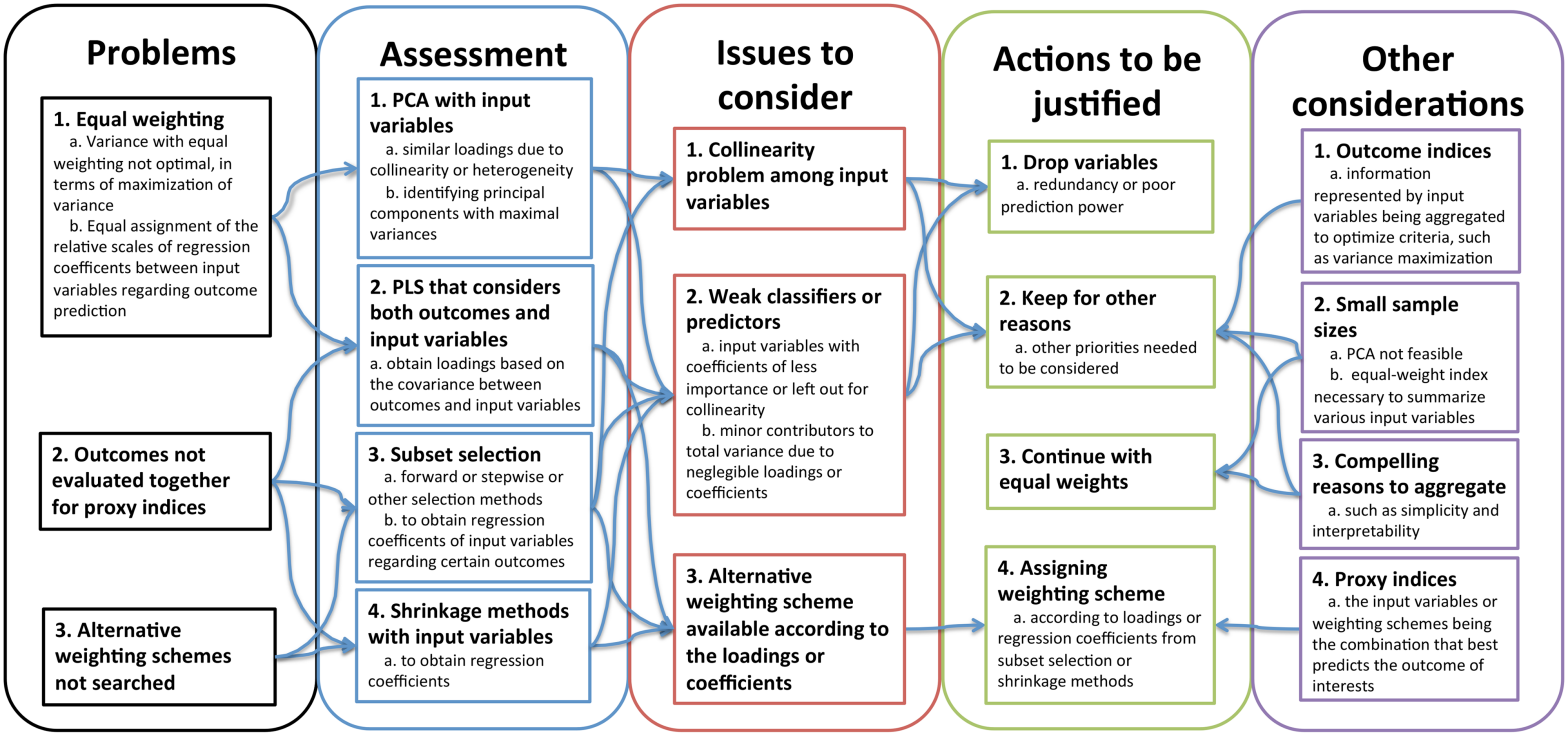
Flowchart of the process of index review and evaluation. Note: PCA: principal component analysis; PLS: partial least squares.

With sufficient sources of publication materials, the focus may soon become how to improve the publication quality or ask authors to comply with review frameworks designed for innovative indices, such as the one we propose in Fig 5. In fact, a standardized reporting guideline should be developed and adopted to review the procedures and justification of index generation. This type of reporting guidelines has been well developed for clinical trials[45], epidemiological studies[46] and systematic reviews[47].

### Proposed framework for the review of innovative indices

To deal with the identified problems and questions to the newly generated mortality indices in a single data set, we suggest the index creators or readers to assess these problems according to research objectives and through analytical methods. Based on our experiences in generating PCA-based indices, it is important to first understand the problems or questions researchers may encounter while mining indices, listed in the Problems box in Fig 5. These questions are related to why imposing equal weights on input variables, whether there are outcomes to be considered, and whether there exists preferred weighting schemes that may be empirical or theory-based.

In the Assessment box in Fig 5, there are tools that can help researchers to understand the weighting schemes and relationships with outcomes, including PCA, PLS transformation, and the regression coefficients obtained from subset selection and shrinkage methods[12]. However, there are other considerations after the initial assessment in the third section, Issues to consider in Fig 5. For example, PCs obtained from PCA can help to address the problem of collinearity. PCA loadings can provide PCA-based weighting schemes and combine weak classifier or insignificant input variables to significant indices. However, the objective of PCA to maximize the PC variances[12] may not be useful if researchers have specific outcomes to consider. Subset selection, such as forward-stepwise regression[12] and random matrix[48], and shrinkage methods, such as LASSO and ridge regression[12], prefer and retain significant input variables. Moreover, nonlinear methods to summarize data, such as non-parametric PCA, diffusion map, and t-SNE (t-distributed stochastic neighbour embedding)[49], are possible options to search for nonlinear projections of input variables.

After reviewing potential problems, assessment results, and important issues in the data set, there are several options toward the data set or weighting schemes, listed in the Actions to be justified section in Fig 5. The first can be the selection of input variables, whether to drop or keep variables. The other is the choice of weighting schemes, equal or unequal weights. If unequal weighting schemes are chosen, it is important to understand the global objectives and the methods to derive the weights, such as PCA or PLS transformation or other projection methods[12].

### Considerations in the evaluation of indices

However, there are other considerations that also matter in the aggregation of information and the generation of index in the last section, Other considerations, in Fig 5. First, whether the new indices will be often referred as outcomes should be considered. The outcome indices can help to represent abstract ideas or concepts. Despite the shortcomings and the necessity to justify the use of equal weights, the outcome indices may be the sums of input variables with equal weights for reasons such as simplicity and interpretability. For example, the number of difficulties in the activities of daily living (ADL) provides understandable and straightforward summaries in functional status, although this adds up the number of difficulties in distinct dimensions, such as bathing and eating[50]. The equal weighting of major dimensions of certain concept, such as functionality, is easy to comprehend and can effectively reduce the number of independent variables to one functional indicator. This is beneficial for studies of small sample sizes. However, there may be alternative weighting schemes much more preferable, if other objectives, such as to maximize aggregate variances or the covariance with the outcomes, exist.

Second, whether the sample sizes of the databases that researcher may use to generate or test new indices are large enough for PCA or other methods is also a key issue. PCA becomes unstable if the number of observations is less than the number of variables[12]. With smaller sample sizes, it is more likely to have PCA be influenced by the outliers in the database and the results of PCA from different databases can vary greatly[12]. Large sample size, universal access and data quality are the reasons why we use the MEPS database to demonstrate the procedures of PC-based index generation and examination.

Lastly, the role of the newly generated indices is also important to consider. For the indices that are treated as outcomes, the theories or existing evidence to combine the indices may be more important than other data objectives. For indices that serve as predictors, proxy indices, the reason why and how to combine input variables are the key to choose the weighting schemes and the methods to generate new indices.

### Limitations

There are several limitations to this study. First, computing power is important for index mining. The creation of a complete matrix of significance in Fig 4 requires more than six-month computing time for a regular desktop computer. Due to this limitation, we are able to test only one outcome, mortality and thus unable to estimate the numbers of indices significant to other outcomes, such as disease incidence and socioeconomic status change.

The weighting and summation of variables based on PCA loadings can lead to a large number of significant weighted indices regarding important outcomes, such as mortality in this study. However, the numbers of publishable indices may be less than those of significant indices due to several reasons. The first is that the adjacent indices produced according to the loadings of the same principal components may be quite similar because some of the loadings can be close to zero. There are currently no methods or algorithms to estimate the exact numbers of publishable indices. We are currently developing several methods to prioritize the significant indices for publication, some of which are computationally intensive. One option is to first examine the significant indices with insignificant neighbouring indices. Another is to use explicit criteria to prioritize indices relative to the neighbouring indices and among the others created based on the same principal components. The criteria can be p values, model fit statistics, or effect sizes regarding specific outcomes. The chosen ones can be those with much lower p values than the neighbouring ones. Other computationally intensive methods we are developing aims to directly interpret the derived indices and select those interpretable and significant ones for publication. This involves algorithms to interpret the derived indices and select based on the similarity between indices and input variables in terms of certain information criteria. The methods to select the best method to aggregate information into indices remains to be further developed and justified.

## Conclusion

PCA loadings can be used to assign weights to input variables and generate innovative indices. With data from 16 longitudinal 2-year MEPS panels, there are 134,689 indices derived from 251 non-redundant variables. Of all indices, there are 40,803 indices significantly associated with mortality in the second years of the MEPS panels with or without the adjustment of age, sex and races. We find that assigning equal weights to variables requires justification and clear objectives. The results help us to develop a preliminary data-driven framework to review the process of index generation. In this framework, the objectives and rationales to combine information from input variables are important issues to consider, as well as the characteristics of the databases. In the face of the possible deluge of innovative indices, we suggest the development of a standard reporting system for the publication of indices and the creation of publication channels for further discussion of information aggregation or variable stacking.

## Supporting information

S1 Table The characteristics of the input variables used to generate principal components. (XLSX)

S2 Table The leading variables contributing the most to the first five principal components. (DOCX)

S1 Equation Discrete-time survival analysis. (DOCX)
