# Supplementary material for: Principal component-based weighted indices and a framework to evaluate indices: Results from the Medical Expenditure Panel Survey 1996 to 2011
Source: PLoS One. 2017 Sep 8;12(9):e0183997. doi: 10.1371/journal.pone.0183997 (PMC5590867; doi:10.1371/journal.pone.0183997)
Supplement: S2 Table — (DOCX) [file pone.0183997.s002.docx]

**S2 Table. The loadings of the first 20 leading variables in the first five principal components.**

|  | PC1 variables | PC1 loadings | PC1 loadings divided by SDs | PC2 variables | PC2 loadings | PC2 loadings divided by SDs | PC3 variables | PC3 loadings | PC3 loadings divided by SDs | PC4 variables | PC4 loadings | PC4 loadings divided by SDs | PC5 variables | PC5 loadings | PC5 loadings divided by SDs |
| --- | --- | --- | --- | --- | --- | --- | --- | --- | --- | --- | --- | --- | --- | --- | --- |
| 1 | ipzeroy1 | -0.0968 | -0.1220 | pubjay1x.1 | -0.1773 | -0.4081 | rxtoty1 | -0.1550 | -0.0681 | uninsy1.1 | 0.2405 | 0.7300 | numemp1 | -0.1929 | -0.0596 |
| 2 | prsdey1.2 | 0.0942 | 0.2663 | pubdey1x.1 | -0.1757 | -0.4003 | totprvy1 | -0.1436 | -0.0303 | inscovy1.3 | 0.2405 | 0.7300 | numemp2 | -0.1912 | -0.0592 |
| 3 | stadey1.2 | 0.0939 | 0.2627 | inscovy1.2 | -0.1570 | -0.4144 | totslfy1 | -0.1403 | -0.0354 | insdey1x.2 | 0.2212 | 0.5962 | stjbyy1 | -0.1861 | -0.0373 |
| 4 | opbdey1.2 | 0.0939 | 0.2632 | numemp1 | 0.1528 | 0.0472 | totmcry1 | -0.1348 | -0.0406 | insjay1x.2 | 0.2174 | 0.5729 | empst1.1 | -0.1772 | -0.3251 |
| 5 | stajay1.2 | 0.0928 | 0.2565 | numemp2 | 0.1514 | 0.0468 | obdprvy1 | -0.1321 | -0.0335 | mcddey1x.1 | -0.1837 | -0.5656 | offer2x.1 | -0.1567 | -0.3215 |
| 6 | prsjay1.2 | 0.0927 | 0.2585 | empst1.4 | -0.1481 | -0.3333 | rxprvy1 | -0.1309 | -0.0361 | mcdjay1x.1 | -0.1773 | -0.5636 | union2.2 | -0.1558 | -0.2926 |
| 7 | obamcdy1 | -0.0926 | -0.1107 | pegdey1.2 | -0.1442 | -0.2964 | obdtchy1 | -0.1276 | -0.0294 | mcdevy1.1 | -0.1748 | -0.4991 | offer1x.1 | -0.1550 | -0.3175 |
| 8 | hhinddy1 | -0.0924 | -0.1109 | pegjay1.2 | -0.1433 | -0.2933 | opdtchy1 | -0.1252 | -0.0490 | uninsy1.2 | -0.1675 | -0.3593 | held2x.1 | -0.1443 | -0.3177 |
| 9 | adlhlp2.2 | 0.0923 | 0.2514 | mcrdey1x.1 | -0.1416 | -0.4032 | opfprvy1 | -0.1251 | -0.0507 | insdey1x.1 | -0.1661 | -0.3356 | actlim1.1 | -0.1236 | -0.4806 |
| 10 | opbjay1.2 | 0.0922 | 0.2546 | stjbyy1 | 0.1399 | 0.0281 | mcrdey1x.1 | -0.1220 | -0.3474 | held2x.2 | 0.1629 | 0.3966 | retpln2.1 | -0.1229 | -0.2831 |
| 11 | pdkdey1.2 | 0.0922 | 0.2566 | totmcry1 | -0.1367 | -0.0412 | optotvy1 | -0.1157 | -0.0929 | offer2x.2 | 0.1613 | 0.4505 | retpln1.1 | -0.1221 | -0.2799 |
| 12 | adlhlp1.2 | 0.0915 | 0.2467 | offer1x.1 | 0.1340 | 0.2745 | insjay1x.2 | 0.1154 | 0.3040 | offer1x.2 | 0.1583 | 0.4463 | hpedey1.1 | -0.1171 | -0.2434 |
| 13 | ipdopuy1 | -0.0915 | -0.1080 | offer2x.1 | 0.1339 | 0.2748 | anylimy1.1 | -0.1151 | -0.2747 | totmcdy1 | -0.1572 | -0.0518 | hpedey1.2 | 0.1150 | 0.2343 |
| 14 | dvomcdy1 | -0.0913 | -0.1075 | evrwrky1.1 | -0.1306 | -0.3610 | ssecpy1x | -0.1151 | -0.0293 | insjay1x.1 | -0.1568 | -0.3123 | hpejay1.1 | -0.1120 | -0.2344 |
| 15 | obnopry1 | -0.0910 | -0.1076 | hpedey1.2 | -0.1305 | -0.2659 | insdey1x.2 | 0.1149 | 0.3095 | obvmcdy1 | -0.1416 | -0.0585 | retpln1.2 | -0.1105 | -0.2743 |
| 16 | pdkjay1.2 | 0.0910 | 0.2504 | hpejay1.2 | -0.1296 | -0.2636 | wrglas2.1 | -0.1136 | -0.2087 | retpln2.2 | 0.1307 | 0.3224 | unable1.1 | -0.1101 | -0.5513 |
| 17 | pogdey1.2 | 0.0908 | 0.2507 | held2x.1 | 0.1294 | 0.2851 | obotchy1 | -0.1134 | -0.0304 | retpln1.2 | 0.1272 | 0.3158 | totmcdy1 | -0.1098 | -0.0362 |
| 18 | obtmcdy1 | -0.0906 | -0.1057 | empst1.1 | 0.1288 | 0.2364 | hpejay1.1 | -0.1134 | -0.2374 | mcdevy1.2 | 0.1227 | 0.2415 | iadlhp1.1 | -0.1092 | -0.6461 |
| 19 | rxofdy1 | -0.0905 | -0.1071 | pubdey1x.2 | 0.1250 | 0.2324 | wrglas2.2 | 0.1124 | 0.2202 | mcdjay1x.2 | 0.1210 | 0.2456 | soclim1.1 | -0.1085 | -0.5407 |
| 20 | rxwcpy1 | -0.0905 | -0.1070 | pubjay1x.2 | 0.1223 | 0.2285 | uninsy1.1 | 0.1123 | 0.3410 | mcddey1x.2 | 0.1198 | 0.2411 | choic2.1 | -0.1071 | -0.2819 |

Note: PC: principal component. See Appendix 1 for variable definitions.
