# Supplementary material for: Principal component-based weighted indices and a framework to evaluate indices: Results from the Medical Expenditure Panel Survey 1996 to 2011
Source: PLoS One. 2017 Sep 8;12(9):e0183997. doi: 10.1371/journal.pone.0183997 (PMC5590867; doi:10.1371/journal.pone.0183997)
Supplement: S1 Equation — (DOCX) [file pone.0183997.s003.docx]

**S2 Equation**. Discrete-time survival analysis.

The outcome, log hazard ratios of mortality in the second years of the MEPS panels, was predicted with information observed in the first years of the MEPS panels.

|  | $\boldsymbol{logit}\left( \boldsymbol{h}_{\boldsymbol{i}}\left( \boldsymbol{t} \right) \right)\boldsymbol{=}{\boldsymbol{\alpha}\left( \boldsymbol{t} \right)\boldsymbol{+}\boldsymbol{\beta}_{\boldsymbol{1}}\boldsymbol{PC}_{\boldsymbol{i}}\boldsymbol{+ \beta}_{\boldsymbol{2}}\boldsymbol{x}}_{\boldsymbol{ji}}\boldsymbol{+ \varepsilon}$  $\boldsymbol{\alpha}\left( \boldsymbol{t} \right)\boldsymbol{=}\boldsymbol{\alpha}_{\boldsymbol{1}}\boldsymbol{Q}_{\boldsymbol{1}}\boldsymbol{+}\boldsymbol{\alpha}_{\boldsymbol{2}}\boldsymbol{Q}_{\boldsymbol{2}}\boldsymbol{+}\boldsymbol{\alpha}_{\boldsymbol{3}}\boldsymbol{Q}_{\boldsymbol{3}}\boldsymbol{+}\boldsymbol{\alpha}_{\boldsymbol{4}}\boldsymbol{Q}_{\boldsymbol{4}}\boldsymbol{+}$  $\boldsymbol{\alpha}_{\boldsymbol{5}}\boldsymbol{Q}_{\boldsymbol{1}}\boldsymbol{PC}_{\boldsymbol{i}}\boldsymbol{+}\boldsymbol{\alpha}_{\boldsymbol{6}}\boldsymbol{Q}_{\boldsymbol{2}}\boldsymbol{PC}_{\boldsymbol{i}}\boldsymbol{+}\boldsymbol{\alpha}_{\boldsymbol{7}}\boldsymbol{Q}_{\boldsymbol{3}}\boldsymbol{PC}_{\boldsymbol{i}}\boldsymbol{+}\boldsymbol{\alpha}_{\boldsymbol{8}}\boldsymbol{Q}_{\boldsymbol{4}}\boldsymbol{PC}_{\boldsymbol{i}}$ | (1)  (2) |
| --- | --- | --- |

In Equation 1, individual log hazard ratios of mortality were modeled with time, denoted by ***t***. Indivisuals were denoted by ***i***.The death time was categorized in four quarters, January to March, April to June, July to September, and October to December (denoted by ***Q_1_*, *Q_2_*, *Q_3_*,** and ***Q_4_***, respectively in equation 2). ***PC_i_*** denoted the PC-based weighted indices for each individual. Only one index was used for each model. Interaction terms between time in quarters and PC-based indices were also included. In the unadjusted models, quarters in the second years of the MEPS panels, PCA-based index and the interaction term were used as independent variables. In the adjusted models, different independent variables, such as age, sex and races, were denoted by ***j***. Error term was denoted by ***ε***.
